# PINNED: identifying characteristics of druggable human proteins using an interpretable neural network

**DOI:** 10.1186/s13321-023-00735-7

**Published:** 2023-07-19

**Authors:** Michael Cunningham, Danielle Pins, Zoltán Dezső, Maricel Torrent, Aparna Vasanthakumar, Abhishek Pandey

**Affiliations:** 1 Genomics Research Center, AbbVie Inc., 1 North Waukegan Rd., North Chicago, IL 60064 USA; 2 Information Research, AbbVie Inc., 1 North Waukegan Rd., North Chicago, IL 60064 USA; 3 Genomics Research Center, AbbVie Inc., 1000 Gateway Boulevard, South San Francisco, CA 94080 USA; 4 Small Molecule Therapeutics and Platform Technologies, AbbVie Inc., 1 North Waukegan Rd., North Chicago, IL 60064 USA

**Keywords:** Protein, Druggability, Machine learning, Neural network, Interpretable, AlphaFold, Protein pocket, Dark genome, Proteome, Drug target

## Abstract

The identification of human proteins that are amenable to pharmacologic modulation without significant off-target effects remains an important unsolved challenge. Computational methods have been devised to identify features which distinguish between “druggable” and “undruggable” proteins, finding that protein sequence, tissue and cellular localization, biological role, and position in the protein–protein interaction network are all important discriminant factors. However, many prior efforts to automate the assessment of protein druggability suffer from low performance or poor interpretability. We developed a neural network-based machine learning model capable of generating druggability sub-scores based on each of four distinct categories, combining them to form an overall druggability score. The model achieves an excellent performance in separating drugged and undrugged proteins in the human proteome, with an area under the receiver operating characteristic (AUC) of 0.95. Our use of multiple sub-scores allows the assessment of potential protein targets of interest based on distinct contributors to druggability, leading to a more interpretable and holistic model to identify novel targets.

**Supplementary Information:**

The online version contains supplementary material available at 10.1186/s13321-023-00735-7.

## Introduction

The cost of developing new therapeutic drugs has risen significantly in recent years, with the average R&D (Research & Development) cost per new drug ranging between $314 million and $2.8 billion [41]. Most of this expense is incurred in the clinical phase, where trial compounds primarily fail due to a poor understanding of the disease process leading to lack of efficacy or toxicity caused either intrinsically by the actual protein being targeted or extrinsically by off-target effects on other proteins [19]. Determining whether a prospective target protein is “druggable,” however, is a complex problem without a clearly understood solution. This can lead to a considerable amount of trial and error in the drug development process. A successful method for pre-screening prospective target proteins for druggability could save billions of dollars per year and increase the number of lifesaving drugs reaching the market.

Druggability is a poorly defined term; it can be used narrowly to refer only to a protein’s ability to bind an activity-modifying small molecule ligand, or more broadly to refer to a protein’s relevance as a therapeutic target in human disease. For this paper’s purposes, druggability encompasses the ability of a protein’s activity to be modulated for pharmacologic effect by a drug which gains regulatory approval. Undruggable proteins are those that cannot be influenced for therapeutic benefit, either because they lack disease relevance, are biologically essential, or cannot be targeted through any known drug modality. Throughout our work we will use these definitions.

Recent advances in machine learning offer the potential for in silico feature identification of druggable proteins. This can facilitate computational evaluation of prospective targets prior to the initiation of expensive clinical trials. A variety of efforts in this area have taken different approaches, incorporating different predictors of druggability into their feature sets. Several groups have sought to solely use properties derived from the primary protein sequence, achieving impressive results in distinguishing drugged proteins from a select subset of difficult-to-drug proteins [17, 21, 26, 28, 35]. However, it is unclear how these models can effectively generalize the entire proteome. Others have analyzed the position of drugged proteins in the protein–protein interaction network to identify common features [14, 27, 29, 40, 43, 46]. Although these models successfully extracted network properties of drugged proteins, their effectiveness is undermined by the lack of information about the protein’s properties, which may be difficult to target chemically.

Given the wide variety of features which may determine whether a protein can be categorized as druggable, it is likely that the most successful approach will incorporate a comprehensive range of properties, including physical and chemical attributes, expression profile, biological functions, and protein–protein interactions. Successful machine learning efforts in this area have utilized features from several of these domains [3, 5, 10, 15, 43]. A 2020 study by Dezső and Ceccarelli focused exclusively on proteins that were targeted by oncology drugs, generating a feature set including a wide variety of chemical, expression, biological function, and network properties. Using a random forest-based model, cancer drug targets were capable of being distinguished from the remainder of the proteome with an AUC of 0.89 [13]. We utilized this feature set and augmented it with additional protein attributes to build a classifier for property identification of drugged human proteins.

To our knowledge, all previously published machine learning models are trained to discriminate druggable from undruggable proteins with a single druggability score or binary classification. This approach lacks interpretability and wholeness, particularly when many distinct types of features are specifically and uniquely contributing to druggability. For example, a protein’s position in the protein–protein interaction network may have major implications for potential off-target effects during clinical trials but does not demonstrate its structural amenability to small molecule modulation. A classifier that separates distinct features into sub-scores prior to obtaining a total druggability score could output multiple types of pertinent information about whether a protein is druggable or undruggable. We created the Predictive Interpretable Neural Network for Druggability (PINNED), a deep learning model which divides its inputs into four distinct groups—sequence and structure, localization, biological functions, and network information—and generates interpretable sub-scores that contribute to a final druggability score.

## Results

Many factors influence a protein’s druggability, including its effectiveness as a disease-modifying target and its propensity for causing undesired side-effects. A protein’s physical and chemical properties, such as amino acid composition, secondary structure, post-translational modification, and others, can determine whether it can be readily liganded by a drug-like molecule. Its position in the complex network of protein–protein interactions which occur within the human body can influence its role in disease and its potential for off-target effects. The biological function of a protein plays a significant role in whether it is a useful drug target; however, many proteins are involved in multiple different processes, disturbance of any can lead to unanticipated consequences for homeostasis and thus leading to off-target effects. Additionally, a protein’s expression profile across target and non-target tissues can have implications for its efficacy and safety.

To incorporate all these contributions to druggability, we generated a feature set that contains a variety of data for 20,404 human proteins, including properties extracted from the protein sequence, tissue specificity, subcellular localization, biological functions, and position in the protein–protein interaction network [13]. The features were divided into four feature groups: sequence and structure, localization, biological functions, and network information. Each category was then augmented with additional features obtained from the protein sequence, Gene Ontology (GO) knowledgebase [1], and the protein’s 3-dimensional structure as estimated by the artificial intelligence system AlphaFold [23] (Table 1).

**Table 1:** All features used to train the model, divided into the four feature groups

Sequence and structure	Localization	Biological functions	Network information
52 physiochemical features	Predicted subcellular localization	Enzyme classification	Signaling maps
Grouped Dipeptide Composition (GDPC)	Tissue specificity	Essentiality of mouse homolog	Network features
Pseudo Amino Acid Composition (PAAC)	Gene Ontology (GO) cellular components	Biological processes (MetaCore)	
fpocket data from AlphaFold models		Molecular functions (MetaCore)	
		Biological processes (Gene Ontology)	
		Gene Ontology (GO) molecular functions	

### Sequence and structure properties

Sequence and structure properties included information about 52 physiochemical features, such as protein molecular weight and amino acid residues, charge and isoelectric points, extinction coefficients, predicted post-translational modifications, secondary structure, and solvent accessibility. Previous works indicate that the grouped dipeptide composition (GDPC) and pseudo amino acid composition (PAAC) of a protein may be useful characteristics in determining its druggability [17, 28, 35]. GDPC represents the relative composition of all the amino acid 2-mers in a protein’s sequence, with the 20 amino acids being reduced to five groups according to their physical properties. PAAC is an algorithm designed to reduce the sequence characteristics of a protein to a defined-length vector while incorporating information about their sequence order [9]. GDPC and PAAC were generated for each of the proteins in our dataset and included in the sequence and structural properties.

AlphaFold is a deep learning network developed by DeepMind that can predict a protein’s structure from its three-dimensional amino acid sequence. The AlphaFold Protein Structure Database was established between AlphaFold and EMBL-EBI [11]. This database contains the predicted protein structure models of accessible UniProt human proteome. It is available as an open-source database. Fpocket is an open-source software package able to automatically detect and provide pocket descriptors in a protein’s 3-dimensional structure [25]. It enables the identification of potential drug binding sites and provides relevant properties based on each pocket detected. The pockets are ranked according to their ability to bind to small molecules as a cavity prediction algorithm. Fpocket was utilized to identify druggable and undruggable protein cavities based on the trajectories produced by the simulation. AlphaFold models of each protein were collected from the AlphaFold database and pocket information was generated using Fpocket.

### Localization

The Subcellular Localization Predictive System (CELLO) was used to predict subcellular localization for each protein in the dataset [44]. We included this prediction, in addition to tissue specificity data obtained from the Genotype-Tissue Expression (GTEx) and the Human Protein Atlas (HPA) [18, 38]. The GO Knowledgebase was used to retrieve Cellular Component annotations for each protein. These labels are manually assigned based on published literature and represent the cellular structures in which the protein performs its functions.

### Biological functions

Gene essentiality, assessed by lethality of mouse homozygous loss-of-function mutations [16] and enzyme classifications obtained from the Swiss-prot database [2], were included in the biological functions score. Scores were generated by Dezső et al. for each gene ontology in the MetaCore database based on their 102-protein target enrichment set of cancer drugs. The highest three ontology scores in the categories—“Biological Functions,” “Molecular Process,” and “Maps” (signaling pathways)—were included in that protein’s feature set. “Biological Functions” and “Molecular Process” were used as inputs to the “biological functions” sub-score, while “Maps” was included in the “network information” sub-score (see below). It should be noted that the Biological Functions score generated by Dezső et al. represents only one feature input into the biological functions network.

### Network information

The signaling pathways (“Maps”) score generated by Dezső et al. was included in the network information features. Degree, closeness, betweenness, eigen centrality, and PageRank of each protein in the protein–protein interaction network were calculated using information from the STRING database [37]. These features were incorporated into the network information input.

**Fig. 1:**
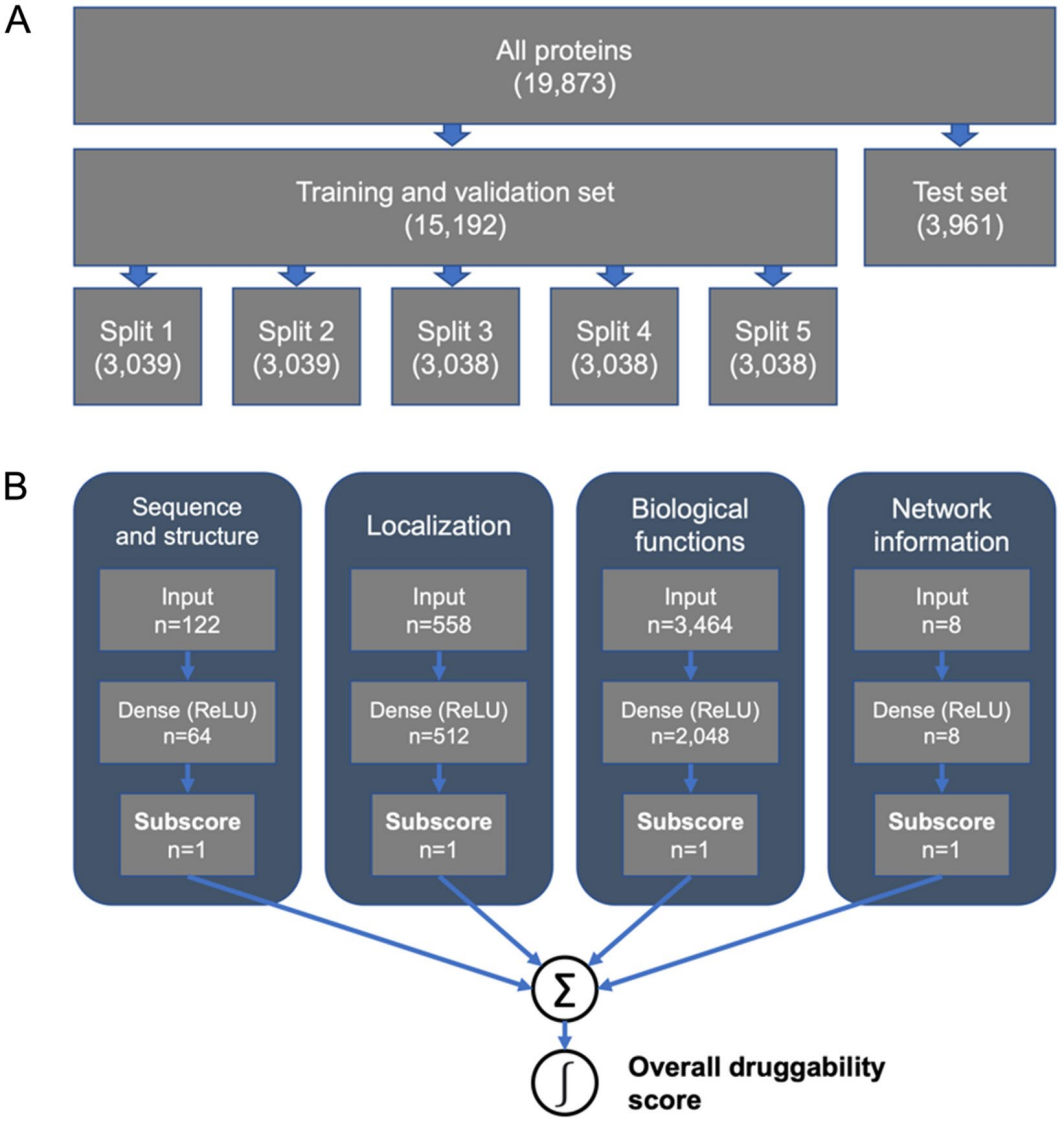
Design of the PINNED model and dataset. **A** Division of the data into training, validation, and test sets. **B** PINNED architecture including the four constituent subnetworks

### Protein set

The National Center for Biotechnology Information (NCBI) Pharos database, a data repository of human protein properties and drugged status, identifies proteins as confirmed drug targets if they are “protein drug targets via which approved drugs act” (“Tclin”) [34]. As of October 2022, 704 of the 20,412 proteins in Pharos are categorized as Tclin.

All other proteins are classified as one of three other categories: undrugged proteins which bind small molecules with high potency (“Tchem”), proteins with well-studied biology (“Tbio”), and proteins not meeting the criteria for any of the other categories (“Tdark”) [34]. Of these proteins, 19,873 were represented in both Dezso et al.’s dataset and the AlphaFold database, including 696 of the 704 Tclin proteins in Pharos. We used the 696 Tclin proteins as our positive “drugged” set, and the remaining 19,177 proteins in the other categories as our negative “undrugged” set (Fig. 1A). It is likely that the undrugged set contains many potentially druggable proteins which have not yet been targeted by approved therapeutics.

### PINNED model

The model architecture consisted of four separate deep neural networks, designated “sequence and structure,” “localization,” “biological functions,” and “network information.” Each network contained an input layer, a hidden layer with ReLU activation, and a single output neuron representing the network sub-score. The four sub-scores were summed, producing a logit which was passed through a sigmoid function to generate the final probability of druggability (Fig. 1B).

Prior to model tuning, 20% of the dataset was held out to form a separate test set, which was used to evaluate the model after the optimal architecture had been determined. The remaining data was divided into five equal groups, one of which was held out as a validation set, while the remaining four were combined to form the training set (5-fold cross-validation) (Fig. 1A). It was necessary to oversample the positive set to prevent the model from converging towards a naïve negative classifier due to the significant imbalance between drugged and undrugged proteins. Within the training set, drugged proteins were separated from the validation set, then randomly oversampled with replacement until the number of drugged and undrugged proteins was equal. The feature matrix was then divided into sequence and structure, localization, biological functions, and network information matrices. These matrices served as inputs to their respective networks.

**Fig. 2:**
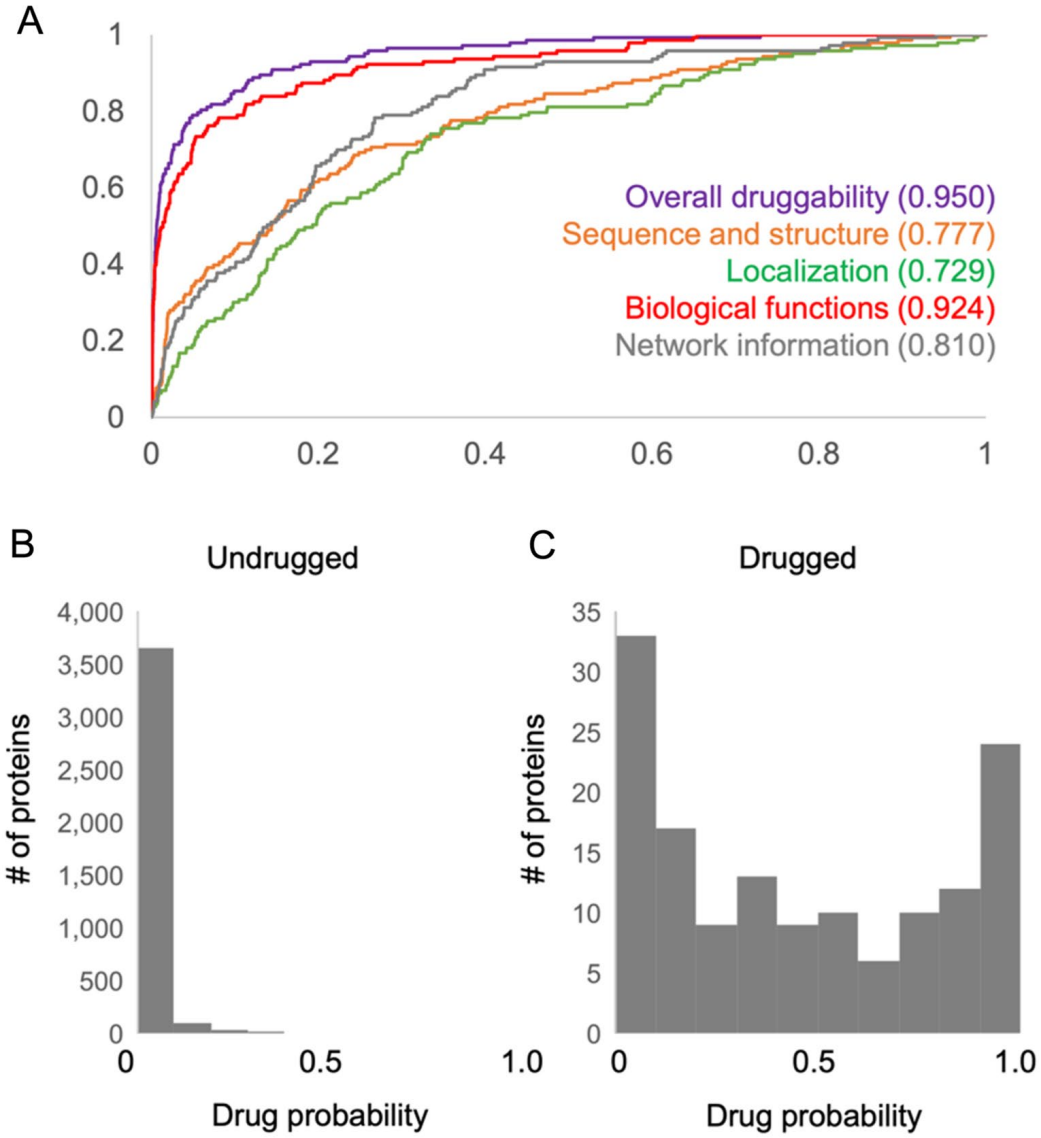
Performance of PINNED on the test set. **A** AUC curve of the model and each subnetwork for distinguishing between drugged and undrugged proteins. **B** Histogram showing the distribution of druggability probabilities for undrugged proteins in the test set. **C** Histogram showing the distribution of druggability probabilities for drugged proteins in the test set

After hyperparameter optimization, a model was trained on the full training/validation set, with the held-out test data used as the final validation set. The complete model achieved an excellent AUC of 0.950 on the test set (Fig. 2A), with the scores from each subnetwork attaining a lower AUC. Although the biological functions sub-score performed by far the best with an AUC of 0.924, the other networks successfully classified proteins as drugged or undrugged with reasonable discriminatory power. The full model consistently scored undrugged proteins in the test set as having low druggability due to the substantial number of negative examples (undrugged proteins) to learn from (Fig. 2B). Druggability scores were more variable for the drugged proteins, reflecting the difficulty of identifying a consistent “druggable” profile from a small number of positives (Fig. 2C). However, PINNED’s high AUC demonstrates its ability to successfully distinguish between proteins with high and low druggability potential.

To determine if the scores could predict success in clinical trials, we tested them against a dataset of successful and failed phase III clinical targets [33]. We found that the overall druggability score achieved an AUC superior to that of the original publication (Additional file 5). Although this may reflect bias in the data, in which more GO annotations or protein–protein interactions have been identified for targets which were successful in clinical trials it indicates that PINNED may be a useful resource for informing not just target selection, but later-stage clinical trials.

**Fig. 3:**
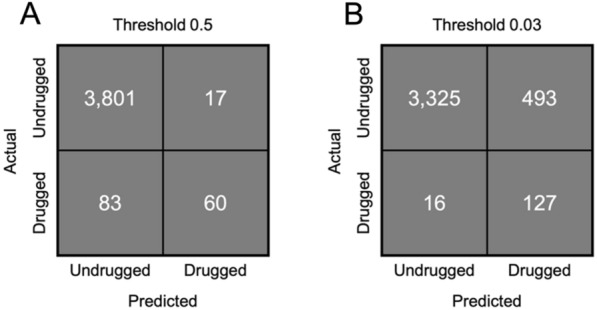
Confusion matrices of PINNED on the test set. **A** Confusion matrix with threshold for druggability set at 0.5. **B** Confusion matrix with threshold set at 0.03 to balance sensitivity and specificity

Reducing the druggability score required to consider a protein “druggable” can increase the sensitivity of the predictor. By default, this value was set as 0.5 during training, but may be changed to any arbitrary value during inference. At a threshold of 0.5, PINNED achieves excellent specificity but low sensitivity, with many drugged proteins in the test set being mistakenly classed as undruggable (Fig. 3A; Table 2). At a reduced threshold of 0.03, chosen to balance sensitivity and specificity, all the drugged proteins are properly classed, while many undrugged proteins are now considered “druggable” (Fig. 3B; Table 2). This cohort of undrugged proteins with high druggability scores represents potential opportunities for pharmaceutical targeting.

**Table 2:** Comparison of PINNED’s test performance at different druggability thresholds

	Sensitivity	Specificity	Accuracy	AUC
0.5 threshold	0.420	0.996	0.975	0.950
0.03 threshold	0.888	0.871	0.871	

A threshold of 0.5 is used for training, while requiring a lower score of 0.03 allows a closer balance between sensitivity and specificity

Comparing PINNED’s performance to prior machine learning efforts to assess protein druggability is challenging due to the wide variety of datasets used and metrics reported. Many previous works exclude proteins with significant homology to drugged proteins from their undrugged sets [3, 14], even though there may be significant differences between these proteins’ properties which alter their utility as drug targets. Similarly, some construct an idealized set of “undruggable” proteins, making it difficult to generalize to the whole proteome [6, 17, 21, 26, 28, 35, 36, 46]. Others only focus on a specific target or indication, such as oncology [4, 12, 13, 22], or ion channels [20]. Restricting our focus to models which seek to assess the druggability of the entire proteome, we find that PINNED comfortably outperforms much of the prior literature in sensitivity, specificity, and AUC [5, 10, 15, 43] (Additional file 1). A recent publication by Raies et al. achieved a higher AUC, but without the constituent sub-scores PINNED generates [32]. The interpretability of our model is a unique advantage which enhances its value to the target selection process.

Of the 696 drugged proteins in our dataset, 294 were affiliated with three protein families: ion channels (124 proteins), G-protein-coupled receptors, or GPCRs (102 proteins), and kinases (68 proteins). The PINNED architecture model of four subnetworks determines which individual members of protein families represent the most promising targets, not just a druggability assessment of entire families. To test the feature learning ability indicating druggability across the whole proteome and apply it to identify targets within unseen protein families, we excluded ion channels, GPCRs, or kinases, respectively, from the training data, and tested the models’ performance on these held-out families. In each case, a training and validation set was constructed consisting of the entire proteome except for the members of the held-out family and used to train five models applying cross-validation. Each model was then implemented to score all proteins within the held-out family, and the scores averaged to generate an ensemble score. The ensemble score AUC in distinguishing drugged and undrugged members of the held-out family was assessed for the overall druggability score and all four constituent sub-scores. This process was repeated for each of the three main drugged families. The PINNED framework maintained the ability to distinguish between drugged and undrugged proteins within each family with reasonable discriminatory power (Table 3). Although the performance of the overall druggability score and each of the sub-networks was reduced relative to a fully heterogeneous training set, the network retained a notable ability to separate drugged from undrugged targets despite not having seen any members of the family in the training data. The sequence and structure sub-score consistently performed poorly, consistent with the same protein family being highly homologous in sequence and in signature structural motifs, and therefore difficult to distinguish. The other three networks incorporate information on each proteins’ known localization, functions, and interactions, respectively, they are more capable of capturing differentiating features which impact druggability, and their performance is correspondingly higher. These results indicate that PINNED can generalize properties of druggable proteins to entire unseen families.

**Table 3:** PINNED performance in distinguishing drugged from undrugged members of major drug target families, as measured by AUC

	Total members (drugged /undrugged)	Overall druggability	Sequence and structure	Localization	Biological functions	Network information
GPCR	400 (102/298)	0.810	0.624	0.662	0.740	0.760
Ion channel	333 (124/209)	0.752	0.544	0.626	0.734	0.662
Kinase	625 (68/557)	0.723	0.518	0.701	0.604	0.845

After training the model and assessing it on the test set, we ablated each feature by randomly shuffling (permuting) the values among the protein test set and assessed the increase in test loss induced by the change. As loss is inversely related to the network’s performance, more prominent features will result in a higher increase in loss after being permuted. We found that features belonging to the biological function’s subnetwork comprised seven of the top 10 (Table 4), consistent both with the substantial number of features in that network and the fact that it was by far the most significant in contributing to PINNED’s performance. Many of the features, including essentiality, degree, transmembrane helices, and PageRank, overlapped with the most notable features selected by Dezső et al. [13]. This indicates a similarity between the properties of oncology targets and other drugged proteins. Additionally, several of the top features derived from GO annotations—ATP binding, voltage-gated potassium channel activity, and potassium ion transmembrane transport—are known to be relevant factors in druggability [8, 42].

**Table 4:** Most notable features, as ranked by change in test loss after random permutation of the feature

	Feature	Category	Change in test loss
1	MetaCore Molecular Function 3	Biological functions	0.00560
2	Enzyme classification—non-enzyme	Biological functions	0.00292
3	Essentiality—unknown	Biological functions	0.00277
4	Degree (STRING interactions)	Network information	0.00232
5	Mitochondrial respiratory chain complex I assembly	Biological functions	0.00208
6	ATP binding	Biological functions	0.00181
7	Transmembrane helices	Sequence and structure	0.00171
8	Voltage-gated potassium channel activity	Biological functions	0.00133
9	PageRank (STRING interactions)	Network information	0.00125
10	Potassium ion transmembrane transport	Biological functions	0.00121

To generate druggability scores for the entire proteome, we split our entire dataset, including the training/validation and test sets into five parts. Each part was held out and the remaining four were used to train a classifier model. The scores for the held-out set were designated as the final druggability scores for the protein set. This process was repeated with each of the sets being held out once to generate scores for the proteins in the entire proteome. Of the 10 highest-scoring undrugged proteins in the proteome, all except TNFRSF11A are listed by Pharos as Tchem, having validated high-potency small molecule ligands (Table 5). The mechanism of action for many drugs is not entirely clear, as they may interact with multiple proteins in the same family, making conclusive classification of proteins as targets or non-targets challenging. We cross-referenced all top 10 scoring proteins with the Therapeutic Targets Database (TTD) and the Open Targets platform, two other databases of drug–target interaction [30, 45]. Of these, five were listed by TTD and two by Open Targets as already being the targets of approved therapeutics, while two were listed by TTD and two by Open Targets as clinical trial targets (Additional file 2). This discrepancy between databases reflects the difficulty of conclusively classifying proteins as mechanism of action drug targets. However, the high prevalence of likely interactors of approved drugs demonstrates that PINNED successfully generalizes the properties of drugged proteins to previously unseen data.

**Table 5:** Highest scoring undrugged proteins

	UniProt ID	Gene name	Protein	Pharos class	Score
1	P21917	DRD4	D (4) dopamine receptor	Tchem	0.9994
2	P50052	AGTR2	Type-2 angiotensin II receptor	Tchem	0.9991
3	Q9Y6Q6	TNFRSF11A	Tumor necrosis factor receptor superfamily member 11 A	Tbio	0.9970
4	P34972	CNR2	Cannabinoid receptor 2	Tchem	0.9969
5	P33032	MC5R	Melanocortin receptor 5	Tchem	0.9967
6	P32241	VIPR1	Vasoactive intestinal polypeptide receptor 1	Tchem	0.9953
7	Q9HCR9	PDE11A	Dual 3′,5′-cyclic-AMP and -GMP phosphodiesterase 11 A	Tchem	0.9953
8	P21918	DRD5	D(1B) dopamine receptor	Tchem	0.9952
9	P23416	GLRA2	Glycine receptor subunit alpha-2	Tchem	0.9927
10	P41968	MC3R	Melanocortin receptor 3	Tchem	0.9925

Of the 20,412 proteins in the Pharos database, 5679 (28%) are designated as “Tdark”—having extremely limited data about their properties and functions. Considerable interest exists in exploring these understudied parts of the genome, particularly to discover novel therapeutic targets which have previously been overlooked [31]. At least one of the top scoring Tdark proteins in our model has been investigated as a drug target (Table 6). Transmembrane protease serine 11B (TMPRSS11B) was identified as upregulated in lung squamous cell carcinomas, serving as a poor prognostic marker. Inhibition of the protein in vitro reduced transformation and proliferation [39].

**Table 6:** Highest scoring Tdark proteins

	UniProt ID	Gene name	Protein	Score
1	Q8TAA3	PSMA8	Proteasome subunit alpha-type 8	0.9277
2	A6NHL2	TUBAL3	Tubulin alpha chain-like 3	0.7725
3	P01880	IGHD	Immunoglobulin heavy constant delta	0.6276
4	Q86T26	TMPRSS11B	Transmembrane protease serine 11B	0.6195
5	Q5TAH2	SLC9C2	Sodium/hydrogen exchanger 11	0.6038
6	Q9Y2U2	KCNK7	Potassium channel subfamily K member 7	0.5257
7	A6NNS2	DHRS7C	Dehydrogenase/reductase SDR family member 7 C	0.4923
8	P0DPH8	TUBA3D	Tubulin alpha-3D chain	0.4891
9	Q5I0G3	MDH1B	Putative malate dehydrogenase 1B	0.4547
10	P01780	IGHV3-7	Immunoglobulin heavy variable 3–7	0.4105

TMPRSS11B’s sub-scores for sequence and structure, localization, biological functions, and network information, compared to the Tclin (drugged) proteins, were respectively in the 84th, 97th, 29th, and 1st percentiles (Additional file 3). The high score for sequence and structure is consistent with the observation that transmembrane helices are highly indicative of druggability (Table 4). Similarly, for the localization subnetwork, permutation importance suggests three of the five most notable features are GO annotations related to localization to the plasma membrane (Additional file 4). Although TMPRSS11B attains a lower score in the biological functions network, it is higher than 95% of undrugged proteins. Its network information score, however, is low even among undrugged proteins, at the 7th percentile. This may indicate that TMPRSS11B lacks the network centrality to have a significant impact on cellular homeostasis. Overall, our results indicate that TMPRSS11B may be structurally amenable to drugging and demonstrates localization and biological activity consistent with other drug targets but may not be indicative of the protein–protein interaction network relative to successfully drugged proteins. The use of multiple sub-scores to characterize a protein’s druggability profile enables a more detailed analysis of its potential strengths and weaknesses rather than a single unified score.

## Discussion

The implementation of a pre-screening methodology that differentiates druggable and undruggable targets can help ameliorate the difficulty of target selection in pharmaceutical development and aid in allocating R&D investments to promising targetable proteins. Consequently, it is imperative that an interpretable model can accurately identify novel druggable targets. We developed a neural network-based machine learning model able to produce druggability sub-scores based on separate feature categories spanning multiple factors in druggability. These allow the analysis of each category individually and its contribution to an overall druggability score.

PINNED attained excellent results in its ability to distinguish drugged from undrugged proteins with an AUC of 0.95. Importantly, this was achieved on the entire proteome, indicating that the model can handle cases generated by family members of drugged proteins. Notably, PINNED was far better at assigning low druggability scores to undrugged proteins than assigning high scores to drugged proteins (Fig. 2), consistent with the large imbalance between the two classes. By reducing the score required to designate a protein as “druggable,” it is possible to increase the sensitivity of the classifier in positively labeling drugged proteins at the expense of also designating as druggable many currently undrugged proteins (Fig. 3). However, these may represent proteins which are already the targets of approved drugs but have not been formally labeled due to insufficient evidence, or potential new targets which merit further investigation (Table 5).

Among our sub-scores, the biological functions network achieved the best performance with a standalone AUC of 0.924. This is potentially due to it being the largest subnetwork, with 3,464 inputs, allowing it to incorporate a large amount of information about protein function. The network information sub-score attained the second-highest performance at 0.810, despite being by far the smallest network, suggesting that the relationship between number of inputs and classification value is complex. Sequence and structure was the lowest-performing subnetwork, achieving an AUC of 0.777 and 0.729. However, these scores are still competitive with previous efforts at using machine learning to assess protein druggability (Additional file 1). This result indicates that our druggability sub-scores are useful not just as inputs to the overall score, but as standalone estimates of each protein’s druggability within that subdomain. Furthermore, we found that PINNED’s overall druggability score exceeds prior publications in predicting success in phase III clinical trials, despite not being trained to directly predict clinical success (Additional file 5).

The 10 most relevant features fed into PINNED, in terms of impact on accuracy, span three of the four subnetworks, with the majority coming from biological functions, but none from localization (Table 4). While this finding is consistent with the fact that the localization subnetwork achieves the lowest standalone AUC, the “transmembrane helices” feature in the sequence and structure network can be assumed to be a strong indicator of whether a protein is localized to the plasma membrane, which dominates the most important localization features (Additional file 4). Some collinearity exists between the feature inputs between the different networks. This is an inevitable result of the proteins’ functions, structures, and interactions being closely interrelated. However, the observation that many proteins score highly on some subnetworks but poorly on others demonstrates that they capture distinct information about a protein’s druggability. Many of the top features overlap with those identified in previous publications [5, 12, 13, 24]. This suggests that machine learning models trained to predict protein druggability converge on a common set of important contributors.

The “dark genome” encompasses the proteins in the human proteome which have not been extensively studied, especially as prospective drug targets, and has thus become of particular interest to the pharmaceutical industry [31]. Our work indicates that a substantial number of proteins in the dark genome may have drug-like properties. For instance, we found transmembrane serine protease TMPRSS11B, a dark genome protein, is similar in structure, localization, and function to many successfully drugged targets. Our model enables dark genome proteins with disease associations to be investigated for druggability potential.

## Conclusions

We established a neural network-based machine learning model, termed PINNED, able to assess proteins’ druggability based on their sub-scores across four distinct categories. We have demonstrated that our proposed methodology is a highly predictive network (test AUC 0.95) with the ability to estimate the druggability of over 20,000 proteins spanning the entire human proteome. PINNED can be used as a pre-screening tool to determine a protein’s amenability to drugging prior to the initiation of pre-clinical programs and identify weaknesses in the form of low sub-scores of top targets that do not necessarily score high in all four areas, providing room for insight and early remediation. This methodology enables the exploration of novel targets cost-effectively while improving the clinical phase success rate.

## Materials and methods

### Drug targets

Drugged and undrugged proteins and sequences were obtained from the Pharos database on October 12, 2022. Proteins categorized as Tclin were labeled as drugged, while proteins categorized as Tchem, Tbio, or Tdark were labeled as undrugged. Protein features were obtained from Dezső et al.’s features [13] and the AlphaFold database [11]. A protein list was generated from the intersection of these three databases. Proteins not found in all the databases were removed, leaving the final protein set used to train the model as the intersection of the three sets. Labels identifying proteins as GPCRs, ion channels, or kinases were obtained from the Knowledge Management Center for Illuminating the Druggable Genome via Pharos on May 20, 2023.

All features generated by Dezső et al. were incorporated into our feature set and divided between the four subnetworks. These include characteristics calculated or predicted from the amino acid sequence, such as posttranslational modifications, enzyme classification, localization, secondary structure, and sequence motifs. Details on the generation of these features can be found in Dezső et al. [13]. All numeric features were standardized to a mean of 0 and standard deviation of 1 (“standard scaled”), while all categorical features were one-hot encoded.

### Sequence and structure properties sub-score

Information about protein molecular weight and amino acid residues, charge and isoelectric points, extinction coefficients, predicted post-translational modifications, secondary structure, and solvent accessibility from Dezső et al.’s feature set were included as sequence and structure properties.

Grouped dipeptide composition (GDPC) and pseudo amino acid composition (PAAC) were calculated using the iFeature toolkit [7]. All selenocysteine (U) residues in the protein sequences were converted to cysteine (C) for the calculations. A lambda of 3 was chosen for PAAC.

Human protein structure predictions were acquired from AlphaFold (last modification on 05/05/2022). The structures were curated to run through Fpocket. Fpocket is an open-source protein prediction algorithm based on the Voronoi tessellation and the alpha sphere theory [25]. Fpocket begins by filtering the vertices and finding the correlated alpha spheres dependent on their minimum and maximum size. Alpha spheres that are clustered together equate to a recognized pocket. The pockets are further reduced based on the zones of compacted atom packing. The alpha spheres are labeled based on their contact to atoms, then ranked based on their prospective binding capabilities towards small molecules. All features were standard scaled.

### Localization sub-score

Protein localization and tissue specificity data obtained from Dezső et al. was included in the localization data.

GO terms were downloaded from the Target Central Resource Database (TCRD) on July 29, 2022, and separated into GO terms categorized as Components, Functions, or Processes. They were used to generate a one-hot encoded GO terms matrix that mapped each protein in the dataset. Terms mapped to less than 10 proteins were excluded. GO Components were included in the localization data, while Functions and Processes were included in the biological functions data (see below).

### Biological functions sub-score

Scores generated for each protein by Dezső et al. from the MetaCore database for “Biological Function,” and “Molecular Process” were standard-scaled and included in the “biological functions” sub-score. The enzyme classification and gene essentiality feature from Dezső et al. were included in the biological functions data.

GO Functions and Processes were obtained and processed as described above and included in biological functions.

### Network information sub-score

The “Maps” (signaling pathways) scores from Dezső et al. and calculated protein–protein interaction network features were used as the input to the network information subnetwork.

### Model

Features for all four sub-scores were combined into a single feature matrix. 20% of the proteins were selected at random prior to model development and held out as a test set. Prior to training, the drugged proteins in the training set were randomly oversampled with replacement until the quantity was equal to the quantity of undrugged proteins. Oversampling by SMOTE, ADASYN, or applying different weights to positive and negative samples were evaluated, but performance was not improved.

Our model was implemented in Python 3.7.13 using TensorFlow 2.11.0 and consisted of four densely connected neural networks, corresponding to the four sub-scores. Each consisted of a single input layer of size *n* inputs, a hidden layer with size 2^*i*^, where *i* is the largest integer such that 2^*i*^ ≤ *n*, and an output layer of size 1, representing that network’s sub-score. ReLU activation was applied to the hidden layers, and an L2 penalty of 0.001 was applied to both the hidden and output layers. The four subnetwork output layers were summed to generate the logits of the overall druggability score. Different numbers of hidden layers, dropout for the input and hidden layers, learning rates, and L2 coefficients were tested, and the above values were found to lead to optimal AUC scores on validation sets.

Support vector machine, logistic regression, XGBoost, and random forest models were also evaluated and found to deliver performance comparable or inferior to neural network.

The model was trained using the Adam optimizer with TensorFlow default parameters at a learning rate of 10^− 3.5^, with a batch size of 32 and the binary cross entropy loss function.

### Supplementary Information

**Additional file 1.** Comparison to previous druggability classifiers.

**Additional file 2.** Target classification of highest scoring undrugged proteins.

**Additional file 3.** All protein scores.

**Additional file 4.** Feature importance scores.

**Additional file 5.** Results from evaluating PINNED scores on phase III clinical targets.

## Data Availability

Our code is available at https://github.com/abbvie-external/Predictive-Interpretable-Neural-Network-for-Druggability-PINNED-.

## Abbreviations

ADASYN: Adaptive Synthetic
AUC: Area Under the Receiver Operating Characteristic
CELLO: Cellular Localization
GDPC: Grouped Dipeptide Composition
GO: Gene Ontology
GPCR: G-protein-coupled receptor
GTEx: Genotype-Tissue Expression
HPA: Human Protein Atlas
NCBI: National Center for Biotechnology Information
PAAC: Pseudo Amino Acid Composition
PINNED: Predictive Interpretable Neural Network for Druggability
R&D: Research and Development
ReLU: Rectified Linear Unit
SMOTE: Synthetic Minority Over-sampling Technique
STRING: Search Tool for the Retrieval of Interacting Genes/Proteins
TCRD: Target Central Resource Database
TMPRSS11B: Transmembrane Serine Protease 11B
TTD: Therapeutic Target Database
