# Supplementary material for: PINNED: identifying characteristics of druggable human proteins using an interpretable neural network
Source: J Cheminform. 2023 Jul 19;15:64. doi: 10.1186/s13321-023-00735-7 (PMC10354961; doi:10.1186/s13321-023-00735-7)
Supplement: Supplementary file 5 — Additional file 5. Results from evaluating PINNED scores on phase III clinical targets. [file 13321_2023_735_MOESM5_ESM.docx]

|  | **AUC** |
| --- | --- |
| **Drug probability** | 0.65 |
| **Sequence and structure** | 0.58 |
| **Localization score** | 0.59 |
| **Biological function score** | 0.62 |
| **Network information score** | 0.52 |

**Supplementary table 5.** Area under the curve (AUC) scores for all PINNED sub-scores in distinguishing successful and failed phase III clinical targets
